# Molar Incisor Hypomineralization in Children with Intellectual Disabilities

**DOI:** 10.3390/dj9020021

**Published:** 2021-02-11

**Authors:** Valentina Brzovic Rajic, Vesna Erika Modric, Ana Ivanisevic Malcic, Kristina Gorseta, Zoran Karlovic, Zeljko Verzak

**Affiliations:** 1 Department of Endodontics and Restorative Dentistry, School of Dental Medicine, University of Zagreb, Gunduliceva 5, 10000 Zagreb, Croatia; vbrzovic@sfzg.hr (V.B.R.); aivanisevic@sfzg.hr (A.I.M.); karlovic@sfzg.hr (Z.K.); 2 Dental Polyclinic Zagreb, Perkovceva 3, 10000 Zagreb, Croatia; vemodric.ordinacija@gmail.com; 3 Department of Paediatric Dentistry, School of Dental Medicine, University of Zagreb, Gunduliceva 5, 10000 Zagreb, Croatia

**Keywords:** dentistry, paediatric dentistry, paedodontics, molar incisor hypomineralization, intellectual disability

## Abstract

The aim of the study is to compare the frequency and the distribution of molar incisor hypomineralization (MIH) in children with intellectual disabilities. Methods: Seventy-two children with intellectual disabilities and 72 healthy children were included in the study. They ranged in age from 5 to 18 years with the same distribution by age and sex. Standard clinical examination was performed, at a dental clinic or in the institution where the children lived, by using a dental mirror and a probe, according the European Academy of Paediatric Dentistry judgment criteria for MIH. Results: Among the 72 children with intellectual disabilities, eight children (11.1%) presented MIH with 19 affected teeth. In the control group, one child (1.4%) presented MIH with two affected teeth. The difference was statistically significant (*p* = 0.033). There were no statistically significant differences between boys and girls. The molars, especially the first right molars were the most affected tooth. Brown defects were less common than white defects. Conclusion: Children with MIH should be identified because this condition is a common problem in children with intellectual disabilities.

## 1. Introduction

Molar incisor hypomineralization (MIH) is defined as enamel hypomineralization of systemic origin of one to four permanent first molars frequently associated with affected incisors [1].

In 2003, a working group of the European Academy of Paediatric Dentistry (EAPD) established judgement criteria for the diagnosis of MIH in epidemiological studies. Each of the permanent first molars and incisors (12 index teeth) should be examined for the presence of demarcated opacities, posteruptive enamel breakdown, atypical restorations (restorations extending to the buccal or palatal smooth surfaces or the incisal third of the crown with opacities adjacent to their margins) and failed eruption of a molar or an incisor. Extracted molars can be considered to have MIH only in cases where there are demarcated opacities or atypical restorations on the other first permanent molars [2,3,4]. At least one permanent first molar should be affected to diagnose MIH [2]. 

An important feature of MIH is the asymmetrical distribution of defects with a marked variation in severity within an individual [1].

The increased porosity of MIH enamel makes the affected teeth sensitive to tooth brushing and other external stimuli, and therefore susceptible to plaque accumulation [5]. The exposed subsurface enamel and dentine may favor bacteria penetration into dentinal tubules. An immunocytochemical study has shown that some non-carious hypomineralized molars have an underlying pulpal inflammation [6]. Restorative treatment of affected teeth is difficult due to an increased sensitivity to water and air blowing, difficulties achieving adequate anaesthesia, and significant dental tissue loss due to either caries or posteruptive enamel breakdown of soft and porous enamel [7,8]. Therefore, dental anxiety and extremely non-cooperative child behavior is fairly common in MIH [9]. In addition, three-fold to 21-fold higher protein content than sound enamel reduces the mechanical properties of enamel [10]. Adhesion of restorative materials to the hypomineralized hard dental tissue is less effective than adhesion to sound enamel [3,11]. Although the demarcated opacities of incisors rarely show enamel breakdown, this sometimes results in aesthetic problems [1].

The MIH worldwide prevalence varies between 2.4% and 40% [12]. The aetiology of this condition remains unknown. However, it has been reported that an alteration in calcium-phosphate balance or insufficient oxygen supply to ameloblasts leads to enamel defects [12]. Some studies have explored the influence of genes and environmental factors on the occurrence of MIH [10,13]. MIH is more common in preterm children [14] and children with systemic diseases. Only 12.2% of children with MIH presented without any relevant medical history [4]. Systemic stress during prenatal or natal period (such as oxygen shortage during birth, hypocalcemia, maternal diseases, Caesarean section) as well as medical or environmental insult within the first three years of life (respiratory diseases, infectious diseases, fever, antibiotics, and dioxins) increase the risk for MIH because the mineralization of enamel in a first permanent molar starts just before birth and is completed by the age of one year [4]. Preterm birth, one of the possible causes of intellectual disabilities, is often associated with medical complications at birth and a child’s immature body functions, both of which may increase the risk of hypoxia [14]. In addition to preterm birth, predisposing factors of intellectual disabilities include genetic factors, infections or intoxications during gestation, as well as perinatal and postnatal events causing central nervous system damage [15]. Some disturbances which alter the neurological development may also affect the development of the tooth germ [16].

The purpose of our cross-sectional study was to determine the frequency and distribution of MIH in children with intellectual disabilities.

## 2. Materials and Methods

A total of 144 children recruited from “houses for children with physical and mental disabilities” in Zagreb, and from four houses for the children without adequate parental care in Zagreb (St. Francis House, the House of Love, Trešnjevka House, and Emaus House) participated in the study from 2012 to 2013. Each group consisted of 72 children aged 5–18 years.

All the children were examined in their institutions by a calibrated examiner, a specialist of paediatric dentistry. The children with intellectual disabilities were examined at the dental clinic in their institution. Tooth surfaces were inspected visually and probed under artificial light, according to the World Health Organization criteria [17]. A plain dental intraoral mirror (Dental mirror SSI-C-003, SS Smile Surgical Ireland Ltd, Limerick, Ireland) without magnification and a blunt probe (SS Smile Surgical Ireland Ltd, Limerick, Ireland) were used. The age, gender, and caries status were recorded for each child. The teeth were cleaned but not dried with compressed air. Number, color, localization, and types of enamel defects (demarcated or diffuse opacities) were recorded using the EAPD judgement criteria [18]. Defects of less than 1 mm in diameter were not recorded [2]. 

Children whose first permanent molars had not erupted or were extracted were excluded from the study, as well as non-cooperative children and children that were not willing to participate in the study. The tooth was considered to be erupted if one-third of the crown was present [19]. The opacities are usually limited to the incisal or cuspal one-third of the crown [3]. The cervical one-third always appears to be sound [11]. Opacities were differentiated from white spot carious lesions based on their texture, demarcation, and their location on teeth and relationship to gingival margin [11,20].

Demarcated opacities have a distinct boundary with the adjacent normal enamel and are white, yellow, or brown [18]. In differential diagnosis, one should consider fluorosis characterized by diffuse opacities, as opposed to the demarcated ones in MIH. Diffuse opacities are white defects that have no clearly defined margin with the adjacent enamel and have a linear, patchy, or confluent distribution.

Opacities affect enamel translucency, but enamel is of normal thickness, while hypoplasia shows reduced localized thickness of enamel. In MIH posteruptive enamel breakdown, the enamel edges are sharp and irregular, while in hypoplasia the borders of the normal enamel are mostly regular and smooth [19].

### Statistical Analyses

Statistical analyses of the data were performed using the package MedCalc Statistical Software version 14.12.0 (MedCalc Software bvba, Ostend, Belgium, http://www.medcalc.org; 2014). The chi-square test was used to compare the prevalence of MIH in the intellectual disabilities group with a control group. The chi-square test or Fisher exact tests were used to access differences in categorical variables between the groups. The level of significance was set at *p* < 0.05. Descriptive statistics were used to present the results. Descriptive data were expressed as mean ± SD or percentages for various parameters, i.e., total number of affected children, total number of affected teeth, and color or shape of affected teeth.

## 3. Results

The study included 144 children aged 5–18 years. Table 1 shows the distribution according to sex in the two groups of children. Sex distribution was not statistically significant between the study group and the control group (X^2^ = 1.400, df = 1, *p* = 0.237).

The mean age at examination was 12.4 (±3.6) years for the children with intellectual disabilities and 12.8 (±3.4) years for the children in the control group, and it did not differ significantly between the two groups of children (*p* = 0.509) (Table 2).

In the group with intellectual disabilities, molar incisor hypomineralization was observed in eight (11.1%) children, i.e., six (75%) boys and two (25%) girls. In the control group, one (1.4%) boy had MIH. The prevalence of MIH in males and females was not significantly different in children with intellectual disabilities (Fisher’s exact test, *p* = 0.702) or in the control group (Fisher’s exact test, *p* = 1.000). 

Children with intellectual disabilities had significantly more MIH defects than children in the control group (Fisher’s exact test, *p* = 0.033).

The most common type of MIH defects were demarcated enamel opacities, present in all children in both groups. Atypical restorations were observed only in the boy in the control group, as well as one tooth extracted due to MIH. There were no children with enamel breakdown.

Diffuse opacities were observed in one (1.4%) child in the control group that was not diagnosed with MIH. 

Nineteen index teeth were affected by MIH in the group of children with intellectual disabilities and twelve teeth (63.2%) of those were the first permanent molars. The majority of opacities in children with intellectual disabilities were on the mandibular right first permanent molar (five teeth). 

**Figure 1:**
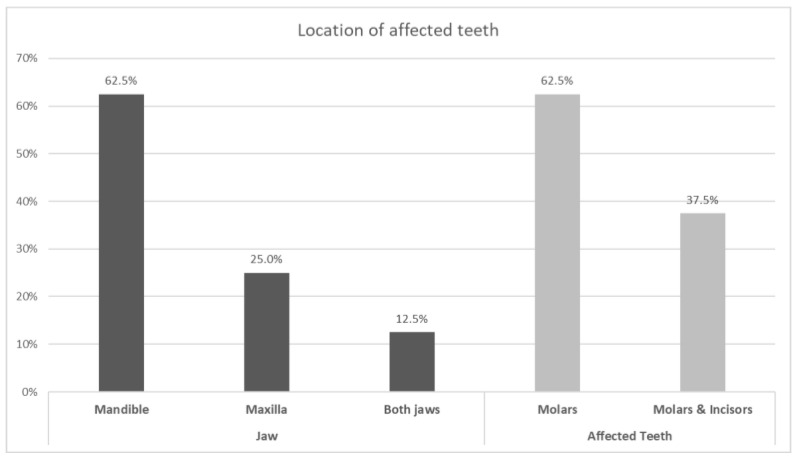
Locations and types of affected teeth.

Characteristics of enamel defects in both groups are listed in Table 3 and the locations of affected teeth are shown in Figure 1.

## 4. Discussion

The decline of caries, in the countries of Western Europe, has increased interest in non-carious lesions, especially MIH that makes the affected teeth prone to caries and its therapy represents a significant challenge to clinicians [4]. Early diagnosis of MIH is important because affected teeth easily crumble and posteruptive enamel loss can lead to rapid caries progression and cause pain [21]. Children with intellectual disabilities often have other medical problems and poor dental health may further compromise their medical condition.

This study found that molar incisor hypomineralization is a significant problem in children with intellectual disabilities affecting 11.1% of children. To the best of our knowledge, MIH has not been investigated in this particular population, however, one study found the developmental defects of enamel (hypomineralization or hypoplasia) prevalence among children with intellectual disabilities was 40.9% [16]; similar to the study conducted by Martínez et al. who reported that 37% of intellectually disabled children had some type of the developmental defects of enamel [22].

In our study, there was no significant difference with regard to sex, which was in contrast to research done by Kemoli [5], Zawaideh et al. [23], Ghanim et al. [24], Glodkowska and Emerich [25], and Ordonez-Romero et al. [26] that concluded molar incisor hypomineralization was more prevalent in girls. However, our study was in agreement with the findings of the following studies: Ng et al. [21], Calderara et al. [27], Jasulaityte et al. [28], Muratbegovic et al. [29], Temilola et al. [30], Koruyucu et al. [31], Buchgraber et al. [32], and Abdalla et al. [33]. The majority of defects in our study were seen on the occlusal and buccal parts of the molar crowns which was in agreement with other studies.

In this research, most defects were asymmetrical. Differences in susceptibility of the ameloblasts during different stages of dental development might explain the asymmetric distribution of MIH defects [34]. An important feature of MIH is the asymmetrical distribution of defects with a marked variation in severity within an individual. This finding is important for distinguishing MIH defects from fluorotic defects that are usually symmetrical, and also from defects such as amelogenesis imperfecta which are more generalized [1]. The tap water in Croatia has low fluoride concentration value and it has never been fluoridated [35], therefore, the low frequency of diffuse opacities in our sample is not surprising.

As shown in Table 3, white defects were more common than brown defects. It has been suggested that the yellow and brown color of the hypomineralized enamel was at a higher risk for posteruptive enamel breakdown and/or atypical restorations as compared with white defects [36] which can be important in tooth prognosis.

The present study showed mandibular molars were affected more than maxillary teeth, which was in contrast to study conducted by Ghanim et al. [24]. Studies conducted by Kemoli et al. [5], Ng et al. [21], Calderara et al. [27], Temilola et al. [30], Buchgraber et al. [32], and Ordonez-Romero et al. [26] have concluded there is no significant difference between the maxilla and mandible teeth with respect to MIH.

A limitation of this study is the age of participants. Eight years of age was recommended as the best time for examination of MIH which should be performed on wet teeth after plaque removal. In most children, at that age, all four first permanent molars should be erupted, as well as the majority of incisors [18], and caries prevalence is still low, therefore, there is a lower risk that enamel defects are masked by carious lesions and tooth colored fillings [28]. However, 33 (45.8%) children in the study group had intact permanent dentition, as well as 24 (33.3%) children in the control group. We could not identify the possible aetiological factors of MIH since children in our sample were living in an institution, therefore, we could not gather, from the parents, information about circumstances of their birth or diseases during the first three years of their children’s lives. That was another limitation of our study, as well as the small sample size.

It is difficult for the children with intellectual disabilities to maintain good oral hygiene by themselves. They have impaired tooth brushing ability and less patience due to their motor, sensor, and learning disabilities [14]. Prevention in the early stage of permanent teeth eruption is crucial. Possible preventive therapies for the treatment of MIH could be beneficial. Some remineralizing agents have been proposed for the treatment of enamel hypomineralization, in addition to conventional fluoride-based therapies, biomimetic hydroxyapatite, and casein phosphopeptide-amorphous calcium phosphate have also been recently introduced and have shown promising results [37,38,39]. Oral health promotion programs should be aimed at centers and schools for children with intellectual disabilities and should include regular use of oral health services, oral hygiene education, and dietary counseling, which is especially important when a child has MIH, a major risk factor for caries.

Future research with more children included is necessary to prove our obtained results. Further studies on MIH prevalence in Croatia and also in other parts of the world are needed in order to investigate the prevalence among the various populations, as well as among children with intellectual disabilities. We also need longitudinal surveys on aetiological factors of these defects.

## 5. Conclusions

Considering the limitations of this study design, it was found that 11.1% of children with intellectual disabilities have molar incisor hypomineralization. Molar incisor hypomineralization is quite common in children with intellectual disabilities. Children with MIH should be identified due to increased caries risk, behavior management problems, and marked reduction in enamel mechanical properties leading to restoration failures. Early intervention should prevent caries development and posteruptive breakdown of MIH defects which would, in turn, prevent dental fear and dental anxiety in children. 

## Figures and Tables

**Table 1:** Gender distribution of examined children.

Gender	Children with Intellectual Disabilities (N/%)	Control Group (N/%)
Male	46 (63.9)	38 (52.8)
Female	26 (36.1)	34 (47.2)
Total	72 (100)	72 (100)

X^2^ = 1.4, df = 1, *p* = 0.237 (there is no gender difference).

**Table 2:** Age distribution of examined children.

Age Group	Children with Intellectual Disabilities (N/%)	Control Group (N/%)
5–7	6 (8.3)	6 (8.3)
8–13	31 (43.1)	36 (50.0)
14–18	35 (48.6)	30 (41.7)

*p* = 0.509.

**Table 3:** Characteristics of enamel defects in examined children.

Characteristics of Enamel Defects	Group of Children with Intellectual Disabilities	Control Group
Number of children affected (*p* = 0.033)	8 (11.1%)	1 (1.4%)
Number of index teeth affected	19 (12 molars, 7 incisors)	2 (2 molars)
Number of affected index teeth per child	2 index teeth, 4 children 1 index tooth, 2 children 3 index teeth, 1 child 5 index teeth, 1 child	2 index teeth
Mean number of affected teeth	2 index teeth	2 index teeth
Location	Jaw, mandible (62.5%) maxilla (25%) both jaws (12.5%) Teeth, only molars (62.5%) molars and incisors (37.5%) Surfaces, buccal (62.5%), occlusal (12.5), palatal (12.5%), buccal and occlusal (12.5%)	Jaw—only maxilla Teeth—only molars Surfaces- Buccal and occlusal
Color	White (87.5%) Brown (12.5%)	Brown
Shape of enamel defects	Asymmetric (75%) Symmetric (25%)	

## References

[1] Weerheijm K.L. (2004). Molar incisor hypomineralization (MIH): Clinical presentation, aetiology and management. Dent. Update.

[2] Lygidakis N.A., Wong F., Jälevik B., Vierrou A.M., Alaluusua S., Espelid I. (2010). Best Clinical Practice Guidance for clinicians dealing with children presenting with Molar-Incisor-Hypomineralisation (MIH): An EAPD Policy Document. Eur. Arch. Paediatr. Dent..

[3] William V., Messer L.B., Burrow M.F. (2006). Molar incisor hypomineralization: Review and recommendations for clinical management. Pediatr Dent..

[4] Lygidakis N.A., Dimou G., Marinou D. (2008). Molar-incisor-hypomineralisation (MIH). A retrospective clinical study in Greek children. II. Possible medical aetiological factors. Eur. Arch. Paediatr. Dent..

[5] Kemoli A.M. (2008). Prevalence of molar incisor hypomineralisation in six to eight year-olds in two rural divisions in Kenya. East Afr. Med. J..

[6] Rodd H.D., Boissonade F.M., Day P.F. (2007). Pulpal status of hypomineralised permanent molars. Pediatr. Dent..

[7] Raposo F., de Carvalho Rodrigues A.C., Lia É.N., Leal S.C. (2019). Prevalence of Hypersensitivity in Teeth Affected by Molar-Incisor Hypomineralization (MIH). Caries Res..

[8] Discepolo K.E., Baker S. (2011). Adjuncts to traditional local anesthesia techniques in instance of hypomineralized teeth. N. Y. State Dent. J..

[9] Jälevik B., Klingberg G.A. (2002). Dental treatment, dental fear and behaviour management problems in children with severe enamel hypomineralization of their permanent first molars. Int. J. Paediatr. Dent..

[10] Hočevar L., Kovač J., Podkrajšek K.T., Battelino S., Pavlič A. (2020). The possible influence of genetic aetiological factors on molar–incisor hypomineralisation. Arch. Oral Biol..

[11] Fagrell T. (2011). Molar incisor hypomineralization. Morphological and chemical aspects, onset and possible etiological factors. Swed. Dent. J. Suppl..

[12] Subramaniam P., Gupta T., Sharma A. (2016). Prevalence of molar incisor hypomineralization in 7–9-year-old children of Bengaluru City, India. Contemp. Clin. Dent..

[13] Teixeira R.J.P.B., Andrade N.S., Queiroz L.C.C., Mendes F.M., Moura M.S., Moura L.F.A.D., Lima M.D.M. (2018). Exploring the association between genetic and environmental factors and molar incisor hypomineralization: Evidence from a twin study. Int. J. Paediatr. Dent..

[14] Brogårdh-Roth S., Matsson L., Klingberg G. (2011). Molar-incisor hypomineralization and oral hygiene in 10-to-12-yr-old Swedish children born preterm. Eur. J. Oral Sci..

[15] Miclea D., Peca L., Cuzmici Z., Pop I.V. (2015). Genetic testing in patients with global developmental delay/intellectual disabilities. A review. Clujul. Med..

[16] Jindal C., Palaskar S., Kler S. (2011). The Prevalence of the Developmental Defects of Enamel in a Group of 8–15 Years Old Indian Children with Developmental Disturbances. J. Clin. Diagn. Res..

[17] Farah R.A., Swain M.V., Drummond B.K., Cook R., Atieh M. (2010). Mineral density of hypomineralised enamel. J. Dent..

[18] Weerheijm K.L., Duggal M., Mejàre I., Papagiannoulis L., Koch G., Martens L.C., Hallonsten A.L. (2003). Judgement criteria for molar incisor hypomineralisation (MIH) in epidemiologic studies: A summary of the European meeting on MIH held in Athens, 2003. Eur. J. Paediatr. Dent..

[19] Ghanim A., Elfrink M., Weerheijm K., Mariño R., Manton D. (2015). A practical method for use in epidemiological studies on enamel hypomineralisation. Eur. Arch. Paediatr. Dent..

[20] Franco K.M., Line S.R., de Moura-Ribeiro M.V. (2007). Prenatal and neonatal variables associated with enamel hypoplasia in deciduous teeth in low birth weight preterm infants. J. Appl. Oral Sci..

[21] Ng J.J., Eu O.C., Nair R., Hong C.H. (2015). Prevalence of molar incisor hypomineralization (MIH) in Singaporean children. Int. J. Paediatr. Dent..

[22] Martínez A., Cubillos P., Jiménez M., Brethauer U., Catalán P., González U. (2002). Prevalence of developmental enamel defects in mentally retarded children. ASDC J. Dent. Child..

[23] Zawaideh F.I., Al-Jundi S.H., Al-Jaljoli M.H. (2011). Molar incisor hypomineralisation: Prevalence in Jordanian children and clinical characteristics. Eur. Arch. Paediatr. Dent..

[24] Ghanim A., Bagheri R., Golkari A., Manton D. (2014). Molar-incisor hypomineralisation: A prevalence study amongst primary schoolchildren of Shiraz, Iran. Eur. Arch. Paediatr. Dent..

[25] Glodkowska N., Emerich K. (2019). Molar Incisor Hypomineralization: Prevalence and severity among children from Nothern Poland. Eur. J. Paediatr. Dent..

[26] Ordonez-Romero I., Jijon-Granja Y., Ubilla-Mazzini W., Porro-Porro L., Alvarez-Giler G. (2019). Distribution of Molar Incisor Hypomineralization in Ecuadorian Children. Dent. Hypotheses.

[27] Calderara P.C., Gerthoux P.M., Mocarelli P., Lukinmaa P.L., Tramacere P.L., Alaluusua S. (2005). The prevalence of Molar Incisor Hypomineralisation (MIH) in a group of Italian school children. Eur. J. Paediatr. Dent..

[28] Jasulaityte L., Veerkamp K.L., Weerheijm K.L. (2007). Molar incisor hypomineralisation: Review and prevalence data from a study of primary school children in Kaunas (Lithuania). Eur. Arch. Paediatr. Dent..

[29] Muratbegovic A., Marcovic M., Ganibegovic S.M. (2007). Molar Incisor Hypomineralisation in Bosnia and: Prevalence, Aetiology and Clinical Consequences in Medium Caries Activity Population. Eur. Arch. Paediatr. Dent..

[30] Temilola O.D., Folayan M.O., Oyedele T. (2015). The prevalence and pattern of deciduous molar hypomineralization and molar-incisor hypomineralization in children from a suburban population in Nigeria. BMC Oral Health.

[31] Koruyucu M., Özel S., Tuna E.B. (2018). Prevalence and etiology of molar-incisor hypomineralization (MIH) in the city of Istanbul. J. Dent. Sci..

[32] Buchgraber B., Kqiku L., Ebeleseder K.A. (2018). Molar incisor hypomineralization: Proportion and severity in primary public school children in Graz, Austria. Clin. Oral Investig..

[33] Abdalla H.E., Abuaffan A.H., Kemoli A.M. (2021). Molar incisor hypomineralization, prevalence, pattern and distribution in Sudanese children. BMC Oral Health..

[34] Fearne J., Anderson P., Davis G.R. (2004). 3D X-ray microscopic study of the extent of variations in enamel density in first permanent molars with idiopathic enamel hypomineralisation. Br. Dent. J..

[35] Mužinić D., Vrček D., IvaniševićMalčić A., Matijević J., RošinGrget K., JukićKrmek S. (2012). The Concentration of Fluorides in tap Water and Commercial Bottled Beverages. Acta Stomatol. Croat..

[36] Da Costa-Silva C.M., Ambrosano G.M., Jeremias F., De Souza J.F., Mialhe F.L. (2011). Increase in severity of molar-incisor hypomineralization and its relationship with the colour of enamel opacity: A prospective cohort study. Int. J. Paediatr. Dent..

[37] Talwar M., Borzabadi-Farahani A., Lynch E., Borsboom P., Ruben J. (2019). Remineralization of Demineralized Enamel and Dentine Using 3 Dentifrices-An InVitro Study. Dent. J..

[38] Scribante A., DermenakiFarahani M.R., Marino G., Matera C., Rodriguez YBaena R., Lanteri V., Butera A. (2020). Biomimetic Effect of Nano-Hydroxyapatite in Demineralized Enamel before Orthodontic Bonding of Brackets and Attachments: Visual, Adhesion Strength, and Hardness in In Vitro Tests. Biomed. Res. Int..

[39] Khanduri N., Kurup D., Mitra M. (2020). Quantitative evaluation of remineralizing potential of three agents on artificially demineralized human enamel using scanning electron microscopy imaging and energy-dispersive analytical X-ray element analysis: An in vitro study. Dent. Res. J..

